# *Mycoplasma hyorhinis* infection promotes gastric cancer cell motility via β‐catenin signaling

**DOI:** 10.1002/cam4.2357

**Published:** 2019-07-18

**Authors:** Xia Liu, Zhuona Rong, Chengchao Shou

**Affiliations:** ^1^ Key Laboratory of Carcinogenesis and Translational Research (Ministry of Education/Beijing), Departments of Biochemistry and Molecular Biology Peking University Cancer Hospital & Institute Beijing China

**Keywords:** gastric cancer cell, motility, *Mycoplasma hyorhinis*, β‐catenin

## Abstract

**Background:**

We previously identified that *Mycoplasma hyorhinis* infection promotes gastric cancer cell motility. The β‐catenin signaling pathway is critical to determining malignant cancer cell phenotypes; however, the association between *M hyorhinis* and the β‐catenin signaling pathway is unclear.

**Methods:**

We performed subcellular fractionation and immunofluorescence staining to observe β‐catenin accumulation in the nucleus. The expression of downstream β‐catenin genes was detected by quantitative RT‐PCR. Gastric cancer cell motility was examined by transwell chamber migration and wound healing assays, and a co‐immunoprecipitation assay was used to detect the proteins associated with the membrane protein p37 of *M hyorhinis*.

**Results:**

We found that *M hyorhinis* infection promoted nuclear β‐catenin accumulation and enhanced the expression of downstream β‐catenin genes. *M hyorhinis*‐promoted gastric cancer cell motility was counteracted by treatment with the β‐catenin inhibitor XAV939 or β‐catenin knockdown. We further detected a protein complex containing LRP6, GSK3β, and p37 in *M hyorhinis*‐infected cells. *M hyorhinis* also induced LRP6 phosphorylation in a GSK3β‐dependent fashion. Knockdown of LRP6 or GSK3β abolished *M hyorhinis*‐induced cell motility.

**Conclusion:**

Our results reveal that the β‐catenin signaling pathway could be activated by *M hyorhinis* infection, thereby contributing to *M hyorhinis*‐induced gastric cancer cell motility.

## 1 BACKGROUND

Gastric cancer is a major public health problem that accounts for approximately 10% of all cancer‐related deaths worldwide. There were more than 900 000 new cases and 700 000 gastric cancer‐related deaths worldwide in 2012.^1^ Many risk factors are involved in the occurrence of gastric cancer, such as the intake of pickled meats or salted vegetables, epigenetic changes, environmental factors, and microorganism infections. The hazards of *Helicobacter pylori* infection in gastric cancer development and progression have been thoroughly investigated; however, the contributions of other microorganisms remain to be determined.

In our previous study, immunohistochemical staining of cancer tissues with a PD4 monoclonal antibody against *Mycoplasma hyorhinis* major membrane protein p37 revealed varying positive rates of *M hyorhinis* infection in several cancer types.^2^ In addition, lymph node metastasis was found to be more pronounced in *M hyorhinis*‐positive cancer tissues.^2^ In gastric cancer and colon cancer, the *M hyorhinis* infection rate was higher in tissues with high differentiation than in tissues with low differentiation,^2^ indicating that *M hyorhinis* is associated with tumor cell malignancy. Namiki et al demonstrated that persistent mycoplasma (*M genitalium* or *M hyorhinis*) infection could promote the chromosomal instability and the malignant transformation of mammalian cells.^3^ Both NF‐κB and EGFR were found to mediate *M hyorhinis*‐induced gastric cancer cell migration.^4^, ^5^ Enhanced phosphorylation of EGFR and ERK1/2 may also contribute to *M hyorhinis*‐induced migration and invasion in vitro and metastasis in vivo.^6^ In *M hyorhinis*‐infected tumor cells, MMP‐2 activity was significantly increased, which in turn stimulated EGFR phosphorylation and contributed to metastasis.^7^ *M hyorhinis* could promote IL‐6 expression via the TLR4 receptor, leading to IL‐6‐mediated STAT3 signaling activation.^8^ Another feature of mycoplasma infection is increased resistance to anticancer agents. Afriat et al^9^ showed that *M fermentans* reduces the activity of TopoI through PARP1 by MAPK activation, thereby altering cellular gene expression and conferring camptothecin resistance. All these studies suggested mycoplasma infection as a possible promoting factor in cancer initiation and progression. However, the signaling pathways mediating mycoplasma‐induced malignant phenotypes have not been fully identified.

The Wnt/β‐catenin pathway, also called the canonical Wnt pathway, is crucial to embryonic development and adult tissue homeostasis. Aberrant activation of the Wnt/β‐catenin pathway results in deregulated cell growth and malignant transformation.^10^, ^11^ This oncogenic pathway is initiated by Wnt, and in the absence of Wnt ligands, cytoplasmic β‐catenin is subjected to phosphorylation via the β‐catenin‐disrupting complex and proteolysis via the ubiquitin‐proteasome system, through which a low level of β‐catenin is maintained.^12^ In the nucleus, transcription factor T‐cell factor/lymphocyte enhancer factor (TCF/LEF) binds to the metastasis inhibitor Groucho/TLE, thereby inhibiting the transactivation of downstream β‐catenin genes.^13^, ^14^ When Wnt binds to receptor LRP6 and Frizzled, it promotes DISH protein aggregation and β‐catenin destruction complex disintegration, thus ensuring β‐catenin accumulation, nuclear entry and downstream gene transcriptional activation.^14^, ^15^ In this study, we provide evidence to show that Wnt/β‐catenin signaling pathway activation contributes to *M hyorhinis*‐induced motility in gastric cancer cells.

## 2 MATERIALS AND METHODS

### 2.1 Reagents and antibodies

Anti‐GSK3β (#22104‐1‐AP), anti‐Wnt2 (#27214‐1‐AP), and anti‐β‐catenin (#51067‐2‐AP) antibodies were purchased from Proteintech (Chicago, IL). Anti‐pSer1490‐LRP6 (#2568) and anti‐LRP6 (#3395) were from Cell Signaling Technology (Beverly, MA). Anti‐β‐tubulin (#ZS‐9104) was purchased from Zhongshan Technology (Beijing, China). Anti‐Histone 2B (#GR193918‐1) was purchased from Abcam (Shanghai, China). Anti‐p37 monoclonal antibody PD4 was generated and characterized previously.^2^, ^5^ FITC‐conjugated and HRP‐conjugated secondary antibodies were from Zhongshan Technology (Beijing, China). XAV939 was purchased from Selleck (Houston, TX).

### 2.2 Mycoplasma propagation and co‐culture

*M hyorhinis* was handled as previously described.^3^, ^4^ Briefly, *M hyorhinis* (Strain #17981, American Type Culture Collection (ATCC)) was cultured for 24 hours at 37°C in modified Hayflick's medium supplemented with 20% heat‐inactivated fetal bovine serum (FBS). Three generations of *M hyorhinis* were cultured continuously. *M hyorhinis* was collected after centrifugation at 12 000 *g* for 20 minutes. After washing once with PBS, *M hyorhinis* was resuspended in PBS and stored at −80°C. We used color change units (CCUs) to calculate the *M hyorhinis* titer. Cells were serum‐starved for 24 hours prior to adding 10^5^ CCU/mL of *M hyorhinis*. *M hyorhinis* inactivation was performed by autoclaving at 121°C and 15 psi for 15 minutes.

### 2.3 Cell culture and siRNA transfection

The human gastric cancer cell lines MGC803 and BGC823 were maintained by Peking University Cancer Hospital & Institute, and the human gastric cancer cell line AGS was obtained from the ATCC. All cells were cultured in RPMI 1640 medium supplemented with 10% FBS in 5% CO_2_ at 37°C. The culture media and FBS were obtained from Invitrogen (Carlsbad, CA). siRNAs (GenePharma, Suzhou, China) were transfected into cells with siRNA‐Mate Reagent (GenePharma). The following siRNAs were used in this study: negative control, 5′‐UUCUCCGAACGUGUCACGUTT‐3′, 5′‐ACGUGACACGUUCGGAGAATT‐3′; GSK3β #1, 5′‐ GCUGGAGUAUACACCAACUTT ‐3′, 5′‐ AGUUGGUGUAUACUCCAGCTT ‐3′; GSK3β #2, 5′‐ GGACAAGAGAUUUAAGAAUTT ‐3′, 5′‐ AUUCUUAAAUCUCUUGUCCTT ‐3′; GSK3β #3, 5′‐ GGACUAUGUUCCGGAAACATT ‐3′, 5′‐ UGUUUCCGGAACAUAGUCCTT ‐3′; LRP6 #1, 5′‐CCACAAAUCCAUGUGGAAUTT‐3′, 5′‐AUUCCACAUGGAUUUGUGGTT‐3′; LRP6 #2, 5′‐GGUUCUGACCGUGUAGUAUTT‐3′, 5′‐AUACUACACGGUCAGAACCTT‐3′; LRP6 #3, 5′‐GCAGAUAUCAGACGAAUUUTT‐3′, 5′‐AAAUUCGUCUGAUAUCUGCTT‐3′; Wnt2 #1, 5′‐CCAGGGUGAUGUGCGAUAATT‐3′, 5′‐UUAUCGCACAUCACCCUGGTT‐3′; Wnt2 #2, 5′‐GCUGGCAGGAAGGCUGUAATT‐3′, 5′‐UUACAGCCUUCCUGCCAGCTT‐3′; Wnt2 #3, 5′‐GCUGACUGGACAACCGCUATT‐3′, 5′‐UAGCGGUUGUCCAGUCAGCTT‐3′; β‐catenin #1, 5′‐CCUUCACUCAAGAACAAGUTT‐3′, 5′‐ACUUGUUCUUGAGUGAAGGTT‐3′; β‐catenin #2, 5′‐GCUCAUCAUACUGGCUAGUTT‐3′, 5′‐ACUAGCCAGUAUGAUGAGCTT‐3′; and β‐catenin #3, 5′‐GUCAACGUCUUGUUCAGAATT‐3′, 5′‐UUCUGAACAAGACGUUGACTT‐3′.

### 2.4 Subcellular fractionation

A total of 5 × 10^6^ cells were collected in 800 μL of buffer A (10 mmol/L HEPES, pH 7.9, 10 mmol/L KCl, 0.1 mol/L EDTA, 1 mmol/L DTT, 1 mmol/L PMSF, and 1 × protease inhibitor cocktail) for 20 minutes on ice, followed by the addition of another 200 μL of buffer A with 0.5% NP‐40. After centrifugation at 1500 *g* and 4°C for 5 minutes, the supernatant was re‐centrifuged at 18 000 *g* and 4°C for 20 minutes to obtain cytoplasmic proteins. The pellet was washed twice with buffer A and homogenized directly in 1× SDS‐PAGE loading buffer to obtain nuclear proteins.

### 2.5 Western blotting

Cells were washed twice with cold PBS and then lysed in buffer containing 25 mmol/L Tris‐HCl pH 7.4, 150 mmol/L NaCl, 1 mmol/L EDTA, 1% NP‐40, 10% glycerol, and 1× protease inhibitor cocktail. After centrifugation at 15,000 *g* for 10 minutes at 4°C, the protein concentrations of the cleared lysates were quantified with a BCA Protein Assay Kit (Thermo Fisher, Waltham, MA). Samples (50 μg per lane) were resolved by 10% SDS‐PAGE, transferred to nitrocellulose membranes, and blocked with 5% non‐fat milk/TBST. Proteins were probed with primary antibodies overnight at 4°C. The membranes were washed three times with TBST and incubated with HRP‐conjugated secondary antibodies for 45 minutes at room temperature and then washed with TBST three times. Signals were detected by enhanced chemiluminescence. For quantification, the optical densities of the protein bands were analyzed by ImageJ software.

### 2.6 Immunofluorescence

Cells were seeded on glass coverslips, grown to 80% confluence, and infected with *M hyorhinis* for 24 hours. Next, the cells were fixed with 2% fresh paraformaldehyde/PBS for 15 minutes at room temperature, washed three times with PBS, permeabilized with 0.5% Triton‐X100/PBS for 20 minutes at room temperature, and blocked with 5% bovine serum albumin (BSA) at room temperature for 1 hour. Rabbit anti‐β‐catenin (1:50; #51067‐2‐AP from Proteintech) was then incubated with the cells overnight at 4°C, followed by washing with PBS three times and probing with a FITC‐conjugated anti‐rabbit antibody for 45 minutes at room temperature in the dark. After washing, cells were counterstained with DAPI (1 μg/mL) for 10 minutes and mounted with 90% glycerol/PBS. A Zeiss LSM780 confocal microscope (Carl‐Zeiss, Oberkochen, Germany) was used to acquire images with fixed settings (60x oil, NA 1.40 Plan‐ApoChromat, including two HyD detectors) and exposure time at room temperature. No Z‐stacking or deconvolution was applied.

### 2.7 Cell migration assay

Cells were starved overnight (12 hours) before the migration experiment. A tissue culture‐treated 6.5‐mm transwell chamber with 8.0‐μm pore membranes (Corning, Corning, NY) was used. Each well of 24‐well plates was filled with 800 μL of medium containing 10% FBS as a chemoattractant. Cells were resuspended in serum‐free medium at a density of 5 × 10^5^ cells/mL, and 150 μL was transferred into the top chamber of each transwell apparatus. The cells were incubated for 24 hours at 37°C. The chambers were then fixed with methanol and stained with hematoxylin. Then, the chambers were washed with water, and the top surface of each membrane was cleared of cells with a cotton swab and counted in five randomly selected microscopic fields per well.

### 2.8 Wound healing assay

Cells were cultured to full confluence in 6‐well plates. The cells were washed with PBS, and wounds were made with sterile pipette tips. Then, culture medium with 5% FBS was added. Images of wound closure at the indicated time points were captured using a Nikon TiU microscope (Nikon, Tokyo, Japan).

### 2.9 Cell proliferation assay

Cells in each group were seeded into 96‐well plates (5 × 10^3^ cells/well) and cultured overnight. Then, the corresponding reagents were added. After 24, 48, and 72 hours, cell proliferation was quantified by cell confluence with a CloneSelect Imager (Molecular Devices, Sunnyvale, CA).

### 2.10 Quantitative RT‐PCR (qRT‐PCR)

Total RNA was isolated from cells using TRIzol reagent (Invitrogen). cDNA was synthesized with a kit from Promega (Madison, WI) according to the manufacturer's protocol. qRT‐PCR was performed using the SYBR premix system (TOYOBO, Osaka, Japan). The sequences of the specific primers were as follows:

β‐catenin, forward primer 5′‐TGCAGTTCGCCTTCACTATG‐3′, reverse primer 5′‐ ACTAGTCGTGGAATGGCACC‐3′; c‐MYC, forward primer 5′‐CCCTCCACTCGGAAGGACTA‐3′, reverse primer 5′‐GCTGGTGCATTTTCGGTTGT‐3′; COX2, forward primer 5′‐GCTCAAACATGATGTTTGCATTC‐3′, reverse primer 5′‐GCTGGCCCTCGCTTATGA‐3′; telomerase reverse transcriptase (TERT): forward primer, 5′‐TCACGGAGACCACGTTTCAAA‐3′, reverse primer, 5′‐TTTCAAGTGCTGTCTGATTCCAAT‐3′; ENAH, forward primer, 5′‐TCTATCACCATACAGGCAACAAC‐3′, reverse primer, 5′‐GCACAGTTTATCACGACCTGA‐3′; GAPDH, forward primer 5′‐TGAAGGTCGGAGTCAACGG‐3′, reverse primer 5′‐CCTGGAAGATGGTGATGGG‐3′. PCR programs were 94°C for 2 minutes, followed by 40 cycles of 94°C for 15 seconds, 56°C for 20 seconds and 72°C for 30 seconds, followed by 72°C for 2 minutes. All gene expression levels were normalized to GAPDH, and the data were analyzed using the 2‐ΔΔCt method.

### 2.11 Statistical analysis

The values represent the mean ± SD of at least three independent experiments with triplicate samples. *P* values were calculated by *t* test using Graph Pad Prism 5. A *P* value less than 0.05 was considered statistically significant.

## 3 RESULTS

### 3.1 *M hyorhinis* infection activates the β‐catenin pathway

**Figure 1:**
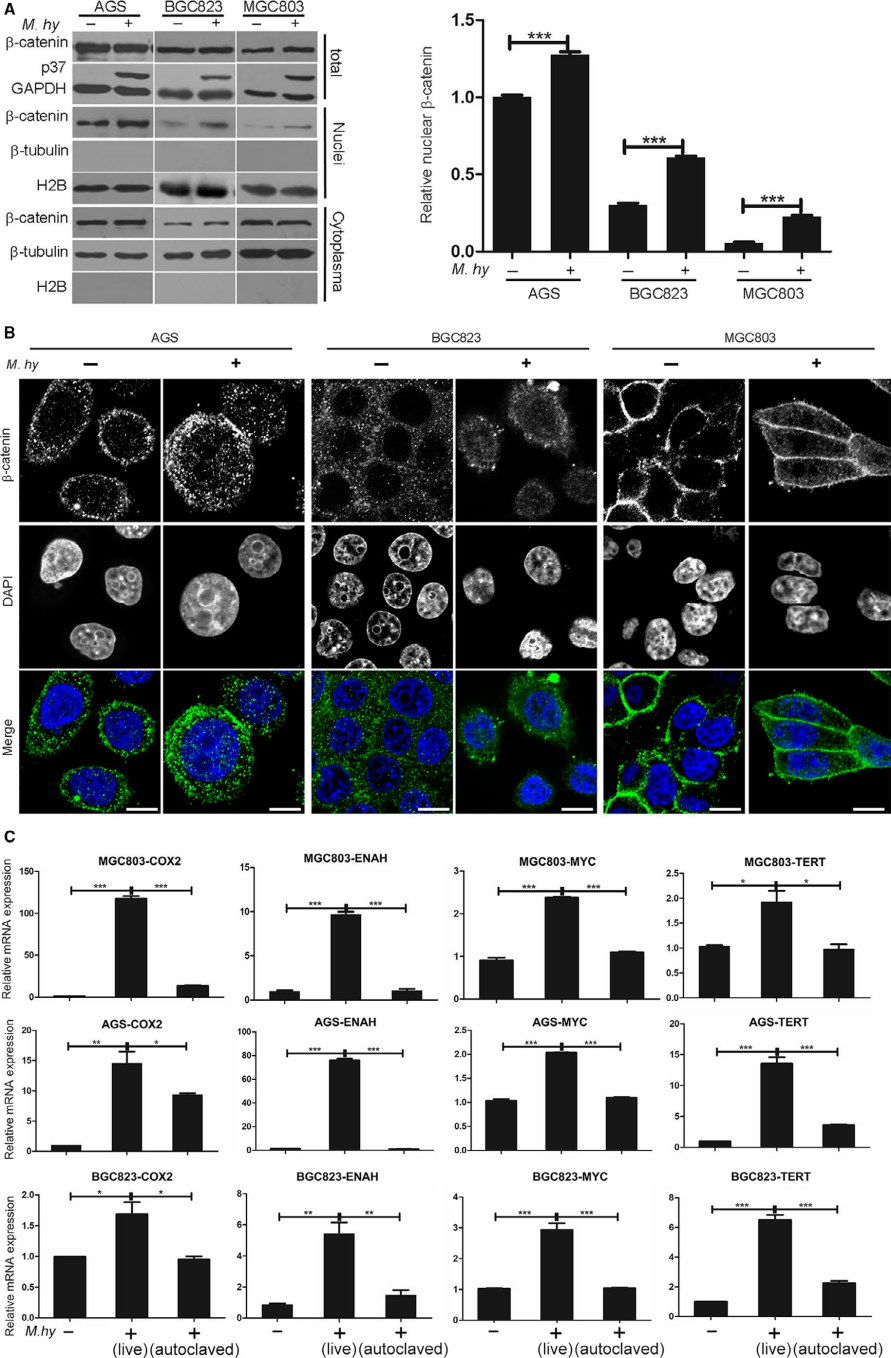
*Mycoplasma hyorhinis* induces β‐catenin translocation into the nucleus and induces the expression of downstream β‐catenin genes. A, (left) Comparison of nuclear β‐catenin in three gastric cancer cell lines with or without *M hyorhinis* infection for 24 h. GAPDH was used as an internal control for total protein, β‐tubulin was used as a cytoplasmic protein marker, and histone 2B (H2B) was used as a nucleoprotein marker. (right) Quantification of the relative expression of nuclear β‐catenin, which was calculated by normalizing to H2B levels. B, β‐catenin (green) localization was observed via immunofluorescence staining after *M hyorhinis* infection for 24 h. Nuclei were counterstained with DAPI (blue). C, qRT‐PCR was performed to detect COX‐2, c‐myc, ENAH, and TERT expression in AGS, BGC823, and MGC803 gastric cancer cells with or without *M hyorhinis* infection for 24 h. As a control, *M hyorhinis* was inactivated through autoclaving. Mean ± SD from three independent experiments. *P < 0.05; **P < 0.01; ***P < 0.001

We first explored the effects of *M hyorhinis* infection on the β‐catenin pathway. Total cell lysates, cytoplasmic proteins and nucleoproteins from *M hyorhinis*‐infected and uninfected gastric cancer cells were analyzed by Western blot. In the total cell lysates, there was no obvious change in β‐catenin levels upon *M hyorhinis* infection. However, nuclear β‐catenin was increased by *M hyorhinis* infection (Figure 1A, left), as confirmed by quantification and statistical analysis (Figure 1A, right). This finding suggested that *M hyorhinis* infection of gastric cancer cells promotes β‐catenin entry into the nucleus. To corroborate this finding, we performed immunofluorescence staining and found that the fluorescence signal of nuclear β‐catenin was markedly enhanced after *M hyorhinis* infection (Figure 1B). We then wondered whether the transcription activity of β‐catenin could be increased following its nuclear relocation. To this end, we carried out qRT‐PCR analysis of four downstream β‐catenin genes, including TERT, which is closely related to cell replication, ENAH, which plays a role in cell motility, c‐myc, an oncogene tightly related to cell transformation, and cyclooxygenase‐2 (COX2), which is a key regulator of the inflammatory response. The mRNA expression levels of these four genes were significantly increased in *M hyorhinis*‐infected gastric cancer cells (Figure 1C). However, such changes were evidently inhibited by autoclave‐induced *M hyorhinis* inactivation (Figure 1C). Therefore, *M hyorhinis* infection activates the β‐catenin signaling pathway.

### 3.2 β‐catenin is required for *M hyorhinis*‐induced gastric cancer cell motility

**Figure 2:**
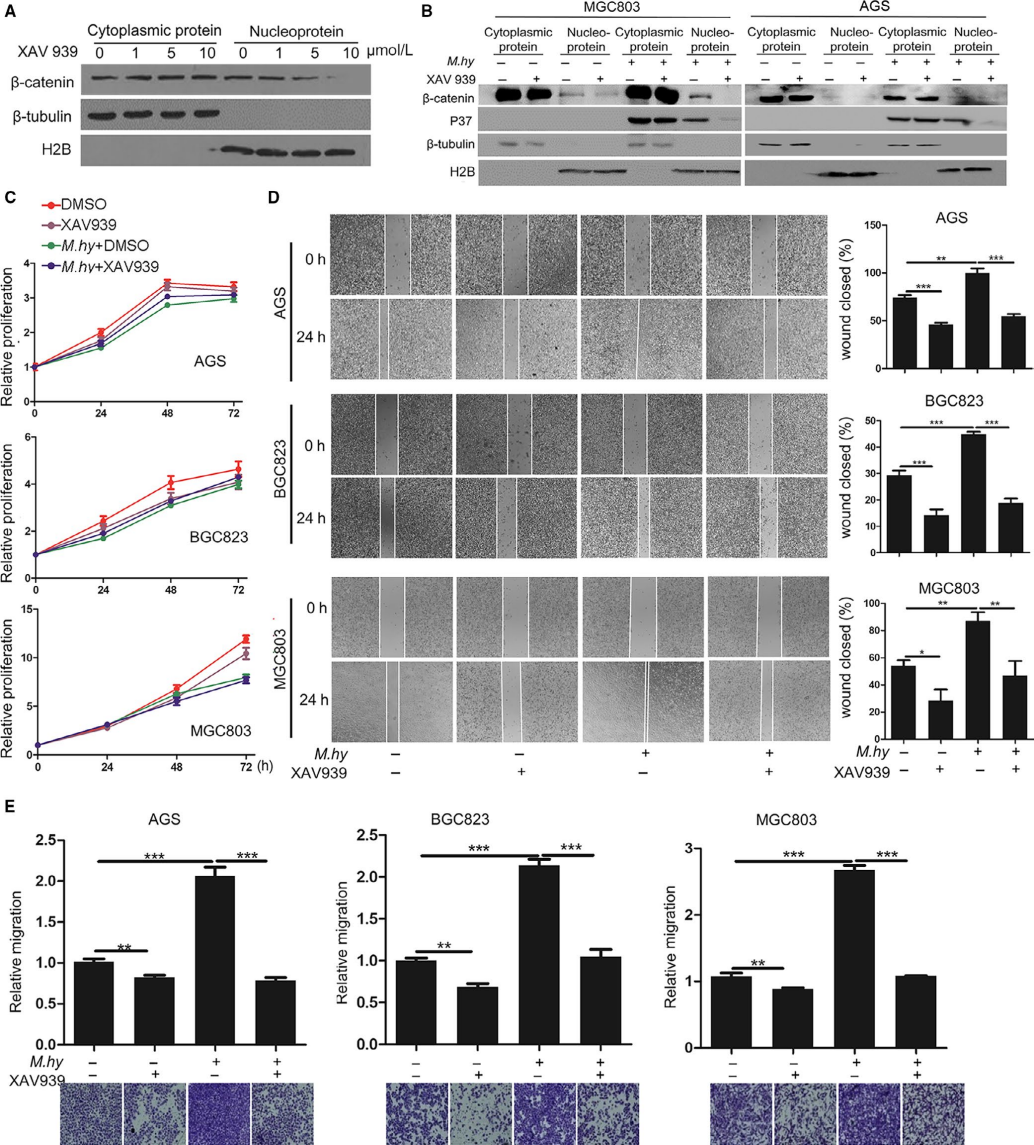
A β‐catenin inhibitor decreased *Mycoplasma hyorhinis*‐induced gastric cancer cell motility. A, XAV939 inhibited nuclear entry of β‐catenin. MGC803 cells were treated with the indicated concentration of XAV939 for 24 h, followed by cytoplasmic and nuclear protein separation. B, XAV939 inhibited *M hyorhinis*‐induced nuclear entry of β‐catenin. MGC803 and AGS cells were pre‐treated with 10 μmol/L XAV939 for 1 hour and infected with *M hyorhinis* for 23 h, followed by cytoplasmic and nuclear protein separation. C, Proliferation of *M hyorhinis*‐infected gastric cancer cell lines in the presence of 10 μmol/L XAV939 or DMSO (vehicle). D, The motility of β‐catenin‐inhibited AGS, BGC823, and MGC803 cells was measured by wound healing assay. Left, representative images of wound healing at the beginning and after 24 h of *M hyorhinis* infection with or without 10 μmol/L XAV939. Right, bar graphs showing the percentages of wound closure. E, The motility of β‐catenin‐inhibited AGS, BGC823, and MGC803 cells was measured by transwell migration assay. Lower, representative images of migrated cells after 24 h of *M hyorhinis* infection with or without 10 μmol/L XAV939. Upper, bar graphs showing the relative migration. Mean ± SD from three experiments with triplicate samples. *P < 0.05; **P < 0.01; ***P < 0.001

**Figure 3:**
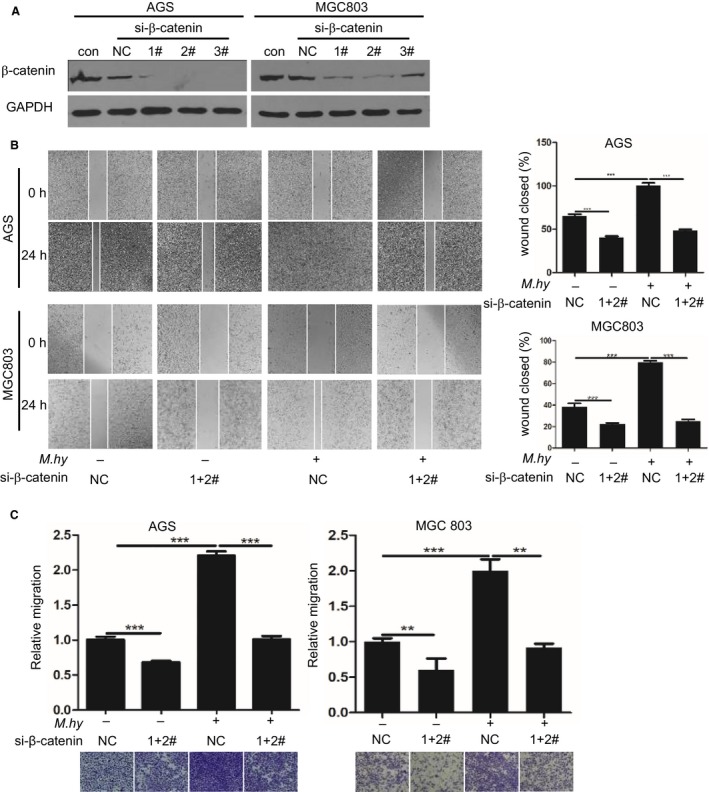
β‐catenin knockdown decreased *Mycoplasma hyorhinis*‐induced gastric cancer cell motility. A, Validation of β‐catenin silencing efficiency. AGS and MGC803 cells were transiently transfected with 50 nmol/L β‐catenin‐specific siRNAs or negative control (NC) siRNA. Forty‐eight hours after transfection, cell lysates were subjected to Western blotting. B, The motility of β‐catenin‐ablated AGS and MGC803 cells was measured by wound healing assay. Twenty‐four hours after siRNA transfection, the cells were re‐plated and subjected to a wound healing assay. Left, representative images of wound healing at the beginning and after 24 h of *M hyorhinis* infection. Right, bar graphs showing the percentages of wound closure. C, The motility of β‐catenin‐ablated AGS and MGC803 cells was measured by transwell migration assay. Twenty‐four hours after siRNA transfection, the cells were re‐plated and subjected to a migration assay. Lower, representative images of migrated cells after 24 h of *M hyorhinis* infection. Upper, bar graphs showing the relative migration. **P < 0.01; ***P < 0.001

To evaluate the contribution of the β‐catenin signaling pathway to *M hyorhinis*‐induced gastric cancer cell motility, we used the β‐catenin‐specific inhibitor XAV939. According to titration, 10 μmol/L XAV939 could efficiently eliminate nuclear β‐catenin (Figure 2A). Pretreatment with this concentration of XAV939 strongly inhibited *M hyorhinis*‐induced nuclear β‐catenin accumulation in both MGC803 and AGS cells (Figure 2B). *M hyorhinis* infection decreased the proliferation of three gastric cancer cell lines (Figure 2C). When we analyzed each time point between cells using the *t*‐test, we found that *M hyorhinis* slightly but significantly inhibited cell proliferation, while XAV939 had minimal effects on basal or *M hyorhinis*‐inhibited proliferation (data not shown). However, XAV939 counteracted *M hyorhinis*‐induced gastric cancer cell motility in wound healing (Figure 2D) and transwell migration assays (Figure 2E). In addition, we utilized three pairs of specific siRNAs to transiently knock down β‐catenin expression (Figure 3A). The stimulatory effect of *M hyorhinis* on cell motility was also counteracted by β‐catenin silencing (Figure 3B and C). These results suggest that β‐catenin is required for *M hyorhinis*‐induced gastric cancer cell motility.

### 3.3 LRP6 and GSK3β mediate β‐catenin pathway activation in *M hyorhinis*‐infected gastric cancer cells

**Figure 4:**
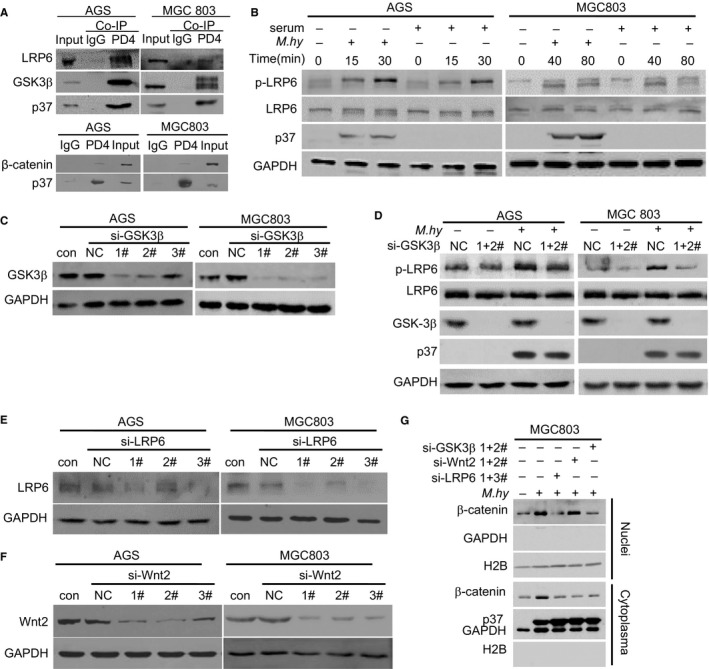
β‐catenin pathway activity was mediated by Wnt2, LRP6, and GSK3β in *Mycoplasma hyorhinis*‐infected gastric cancer cells. A, Co‐immunoprecipitation assay to validate the physical association of LRP6, GSK3β, and the p37 protein of *M hyorhinis*. Lysates from *M hyorhinis*‐infected gastric cancer cells (500 μg) were immunoprecipitated with 1 μg of PD4 antibody or pre‐immune IgG. Input, 50 μg of lysates. B*, M. hyorhinis* upregulated LRP6 phosphorylation in gastric cancer cells. Cells were serum‐starved for 24 h and then infected with *M hyorhinis* or stimulated with serum. Cell lysates were harvested at the indicated time points and analyzed by Western blotting. C, Validation of GSK3β silencing efficiency. Cells were transfected with 50 nmol/L GSK3β‐specific siRNAs or NC siRNA for 48 h. D, Knocking down GSK3β expression downregulated *M hyorhinis*‐induced LRP6 phosphorylation. Cells were transfected with the indicated siRNAs for 48 h and then infected with *M hyorhinis* for 30 min. E, Validation of LRP6 silencing efficiency. Cells were transfected with 50 nmol/L LRP6‐specific siRNAs or NC siRNA for 48 h. F, Validation of Wnt2 silencing efficiency. Cells were transfected with 50 nmol/L Wnt2‐specific siRNAs or NC siRNA for 48 h. G, Effect of GSK3β, Wnt2, or LRP6 ablation on *M hyorhinis*‐induced nuclear entry of β‐catenin. Cells were transfected with the indicated siRNAs for 24 h and infected with *M hyorhinis* for 24 h, followed by cytoplasmic and nuclear protein fractionation

Interestingly, an immunoprecipitation assay with a PD4 antibody against the p37 protein detected a complex containing p37 and two upstream regulators of β‐catenin signaling, including GSK3β and the Wnt receptor LRP6,^16^, ^17^, ^18^ in *M hyorhinis*‐infected gastric cancer cells (Figure 4A). Furthermore, *M hyorhinis* infection quickly increased LRP6 phosphorylation (Figure 4B). When GSK3β expression was ablated by siRNA‐mediated knockdown (Figure 4C), *M hyorhinis*‐induced p‐LRP6 was decreased (Figure 4D), suggesting that LRP6 phosphorylation is GSK3β‐dependent in the context of *M hyorhinis* infection. This conclusion is also consistent with the role of GSK3β as a kinase inducing LRP6 phosphorylation.^16^, ^17^ We also decreased LRP6 (Figure 4E) or Wnt2 (Figure 4F) expression by siRNA and found that *M hyorhinis*‐induced nuclear β‐catenin accumulation was attenuated by either LRP6 or GSK3β ablation (Figure 4G). On the other hand, the inhibitory effect of Wnt2 knockdown on *M hyorhinis*‐induced nuclear β‐catenin accumulation was relatively minimal (Figure 4G), indicating that GSK3β and LRP6, but not Wnt2, are essential for β‐catenin activation upon *M hyorhinis* infection.

**Figure 5:**
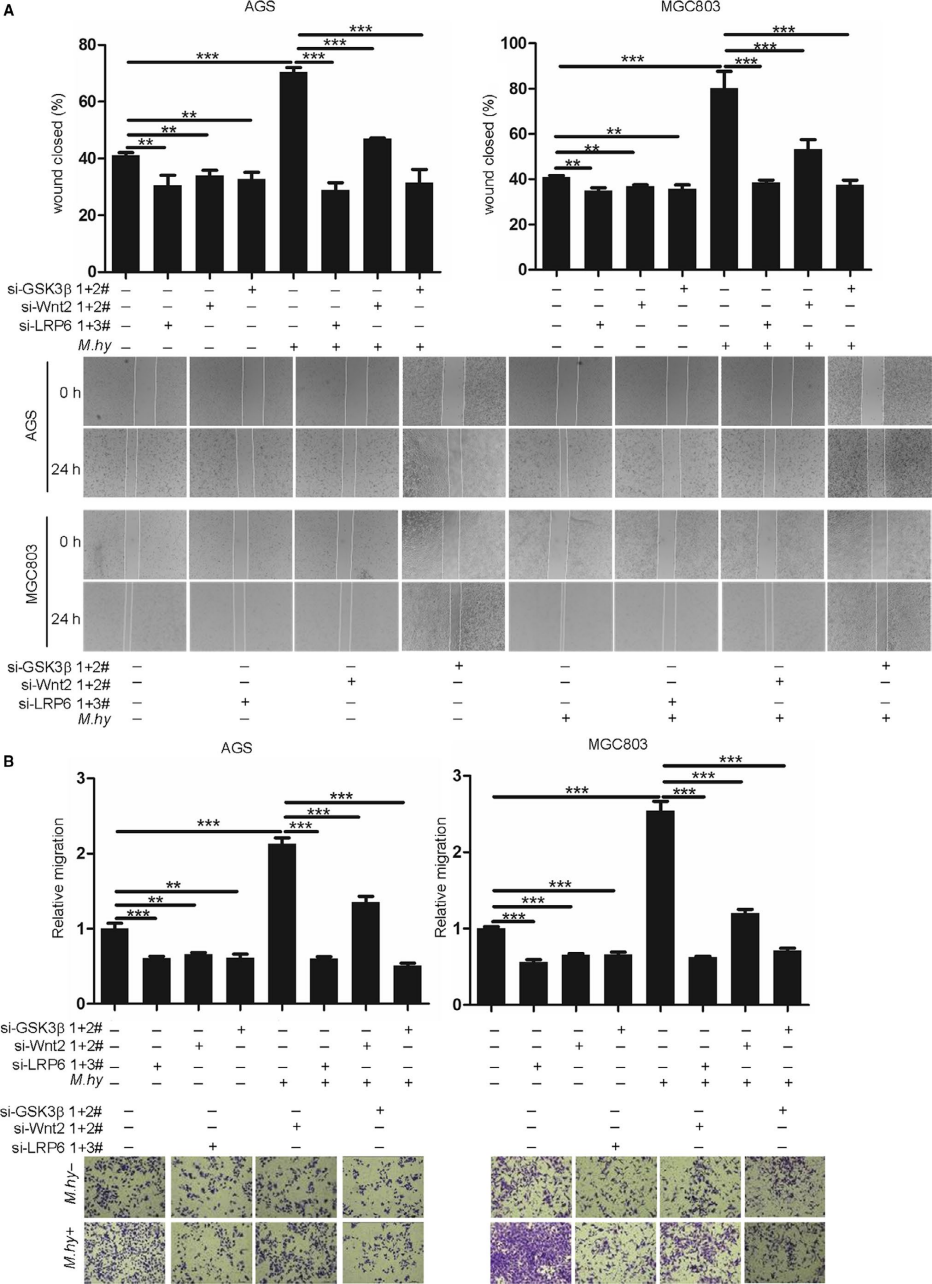
GSK3β, LRP6, and Wnt2 knockdown decreased *Mycoplasma hyorhinis*‐induced gastric cancer cell motility. A, The motility of GSK3β‐, LRP6‐, or Wnt2‐ablated AGS and MGC803 cells was measured by wound healing assay. Twenty‐four hours after siRNA transfection, the cells were replated and subjected to a wound healing assay. Lower, representative images of wound healing at the beginning and after 24 h of *M hyorhinis* infection. Upper, bar graphs showing the percentages of wound closure. B, The motility of GSK3β‐, LRP6‐, or Wnt2‐ablated AGS and MGC803 cells was measured by transwell migration assay. Twenty‐four hours after siRNA transfection, the cells were re‐plated and subjected to a migration assay. Lower, representative images of migrated cells after 24 h of *M hyorhinis* infection. Upper, bar graphs showing the relative migration. Mean ± SD from three independent experiments. **P < 0.01; ***P < 0.001

Next, we performed wound healing (Figure 5A) and migration assays (Figure 5B) and found that GSK3β, LRP6, or Wnt2 knockdown significantly abolished *M hyorhinis*‐induced gastric cancer cell motility, but the effect of Wnt2 ablation was less robust than that of the other two factors. Thus, Wnt2, as a ligand of LRP6 in β‐catenin signaling, has a less important role in *M hyorhinis*‐induced gastric cancer cell motility.

## 4 DISCUSSION

Wnt/β‐catenin pathway dysfunction plays an important role in the development of cancer and other diseases. An adenomatous polyposis coli (APC) mutation was detected in patients with familial adenomatous polyposis (FAP) and in sporadic colon cancer.^19^, ^20^ Nuclear β‐catenin accumulation was found to be associated with APC mutation in colon cancer.^21^ In addition, stabilizing β‐catenin mutations were identified in melanoma and colon cancer cell lines,^22^, ^23^ and Axin1 and Axin2 mutations were associated with colon cancer.^24^, ^25^ Deregulated expression of other factors of the β‐catenin pathway, including sFRP1 silencing, Wnt overexpression, and LRP5 internal deletion, were also implicated in carcinogenesis.^26^, ^27^, ^28^, ^29^, ^30^, ^31^

In this study, we revealed that the β‐catenin pathway plays an important role in *M hyorhinis*‐induced gastric cancer cell motility. Our data demonstrated that the β‐catenin pathway is activated by *M hyorhinis* infection and contributes to *M hyorhinis*‐induced cell motility, possibly by transactivation of downstream β‐catenin genes. We further found that GSK3β and LRP6 are essential for *M hyorhinis*‐induced β‐catenin activation, while the role of Wnt2 is less important. The monoclonal clonal antibody PD4 specifically recognizes lipoprotein p37,^32^ which is the most abundant membrane protein and the major antigen of *M hyorhinis*.^33^ The results from several groups, including our lab, found that p37 has several roles in different stages of tumorigenesis through coupling with multiple signaling pathways.^5^, ^7^, ^34^, ^35^ Based on these considerations, we used a PD4 mAb to examine the possible association between p37 and factors involved in the Wnt signaling pathway. We detected a physical interaction between LRP6 and p37; thus, it is possible that p37 may bypass the role of Wnt2 ligand and activate β‐catenin signaling through LRP6. However, this hypothesis needs to be verified in future studies. We also noted that *M hyorhinis* infection caused greater LRP6 phosphorylation in AGS cells than in MGC803 cells. It is likely that the responsiveness of different cells to *M hyorhinis* infection could be different, and the kinetics of LRP6 phosphorylation in different cells could also be distinct.

In the Wnt/β‐catenin pathway, the serine/threonine protein kinase GSK3β functions as a negative regulator of β‐catenin.^15^, ^18^ Despite this negative role, GSK3β also possesses the activity to phosphorylate LRP6, thereby positively regulating the Wnt/β‐catenin pathway.^16^, ^18^ In vitro, GSK3 binds directly to LRP6 ICD and phosphorylates PPSP repeats.^16^, ^17^ When GSK3β expression was inhibited by siRNA, we noticed that the amount of *M hyorhinis*‐induced p‐LRP6 was decreased. This result further indicates that *M hyorhinis* can promote β‐catenin signaling through the GSK3β‐LRP6 pathway. Because the β‐catenin signaling pathway regulates multiple malignant cancer cell phenotypes,^10^, ^11^ the possibility that *M hyorhinis*‐induced β‐catenin activation may affect other aspects of tumorigenesis warrants further investigation.

Cross‐talk between the β‐catenin pathway and other pathways has been reported. For example, *Helicobacter pylori*‐induced activation of EGFR‐PI3K/Akt signaling resulted in GSK3β suppression and β‐catenin accumulation through VacA or OipA.^36^, ^37^, ^38^ We previously reported that *M hyorhinis* infection activated the EGFR‐PI3K/Akt signaling axis in gastric cancer cell lines.^5^ Therefore, it will be interesting to examine the association between the β‐catenin pathway and the EGFR‐PI3K/Akt pathway. It should be noted that EGFR also plays a critical role in mediating *M hyorhinis* infection, likely through physical association with p37 protein.^4^, ^5^ However, LRP6, GSK3β, or Wnt2 knockdown did not affect p37 levels (Figure 4D and G), despite the fact that p37 could complex with LRP6 and GSK3β (Figure 4A). Therefore, LRP6, GSK3β, and Wnt2 are dispensable for *M hyorhinis* infection.

We next detected p37's associations with GSK3β and LRP6. *M hyorhinis*‐induced nuclear β‐catenin accumulation was impaired by LRP6 or GSK3β knockdown, while the effect of Wnt2 knockdown was minimal (Figure 4G). The wound healing and migration assay results showed that LRP6 or GSK3β knockdown completely abolished *M hyorhinis*‐induced motility, but the effect of Wnt2 knockdown was partial (Figure 5). We therefore proposed that *M hyorhinis*‐stimulated β‐catenin pathway activation is dependent mainly on LRP6 and GSK3β, whereas Wnt2's contribution is relatively smaller.

The p37 protein is the most abundant membrane protein of *M hyorhinis* 33; thus, it is possible that p37 is the main promoter of Wnt‐β‐catenin via its interactions with LRP6 and GSK3β. However, at present, this remains speculation because there is no direct evidence available. We did try to block p37 and determine changes in the Wnt‐β‐catenin pathway; however, due to the critical role of p37 in mediating *M hyorhinis* infection,^4^ the result was inconclusive (data not shown). Although purified p37 had a strong capacity to bind cultured cells and activate the EGFR or NF‐κB pathway,^4^, ^7^ preliminary results showed that the p37 protein had low activity to induce LRP6 phosphorylation and nuclear β‐catenin accumulation (data not shown). It is possible that p37 requires the assistance of other factor(s) from host cells or/and *M hyorhinis* to fully induce β‐catenin signaling, which will be explored in future studies.

Nevertheless, by identifying the contributing role of β‐catenin, as well as LRP6 and GSK3β, in *M hyorhinis*‐induced motility, this study may provide valuable evidence for patient‐directed therapies for *M hyorhinis*‐positive gastric cancer patients.

## 5 CONCLUSION

We found that the β‐catenin signaling pathway is activated by *M hyorhinis* infection. Targeting the key factors in the β‐catenin signaling pathway, including β‐catenin, GSK3β, and LRP6, could counteract *M hyorhinis*‐induced gastric cancer cell motility.

## ETHICS APPROVAL AND CONSENT TO PARTICIPATE

Not applicable.

## CONSENT FOR PUBLICATION

Not applicable.

## CONFLICT OF INTERESTS

The authors declare that they have no competing interests.

## AUTHORS' CONTRIBUTIONS

CS conceived the project. CS and XL designed the experiments. XL performed the experiments. CS and XL analysed the data. XL wrote the manuscript. All authors read and approved the final manuscript.

## Data Availability

All the data supporting our findings can be found in the Results section of the paper.
